# The *CARL II* cohort: what we did and what came out of it

**DOI:** 10.1186/s12998-026-00641-3

**Published:** 2026-04-17

**Authors:** Jan Hartvigsen, Sasha Aspinall, Aron Downie, Steen Harsted, Hazel Jenkins, Andrée-Anne Marchand, David McNaughton, Amy Miller, Casper Nim, Luana Nyirö, Eric J. Roseen, James J. Young, Kenneth A. Weber, Jessica J. Wong, Cecilie K. Øverås, Diana De Carvalho, Jon Adams

**Affiliations:** 1 Department of Sports Science and Clinical Biomechanics, Center for Muscle and Joint Health, University of Southern Denmark, Odense M, Denmark; 2 Chiropractic Knowledge Hub, Odense M, Denmark; 3 Danish Institute for Advanced Study, Odense M, Denmark; 4 School of Allied Health, Murdoch University, Perth, Australia; 5 Department of Chiropractic, Faculty of Medicine, Health and Human Sciences, Macquarie University, Sydney, Australia; 6 Macquarie University Spinal Pain Research Centre, Macquarie University, Sydney, Australia; 7 SDU Health Informatics and Technology, The Maersk Mc-Kinney Moller Institute, Odense M, Denmark; 8 Department of Chiropractic, Université du Québec à Trois-Rivières, Trois-Rivières, Québec Canada; 9 School of Health, Medical and Applied Sciences, College of Health Sciences, Central Queensland University, Brisbane, Australia; 10 Health Sciences University, Bournemouth Campus, Bournemouth, UK; 11 Medical Research Unit, Spine Centre of Southern Denmark, University Hospital of Southern Denmark, Kolding, Denmark; 12 Department for Regional Health Research, University of Southern Denmark, Odense, Denmark; 13 Department of Chiropractic Medicine, Balgrist University Hospital, University of Zurich, Zurich, Switzerland; 14 Department of Medicine, Section of General Internal Medicine, Boston University Chobanian & Avedisian School of Medicine and Boston Medical Center, Boston, MA USA; 15 Schroeder Arthritis Institute, University Health Network, Toronto, Canada; 16 Department of Anesthesiology, Perioperative and Pain Medicine, Stanford University School of Medicine, Palo Alto, CA USA; 17 Faculty of Health Sciences, School of Physical Therapy, Western University, London, ON Canada; 18 Department of Epidemiology and Biostatistics, Schulich School of Medicine & Dentistry, Western University, London, ON Canada; 19 Department of Public Health and Nursing, Faculty of Medicine and Health Sciences, Norwegian University for Science and Technology, Trondheim, Norway; 20 Norwegian Chiropractor’s Research Fund – Et Liv I Bevægelse (ELiB), Oslo, Norway; 21 Division of Population Health and Applied Health Science, Faculty of Medicine, Memorial University of Newfoundland, St. John’s, NF Canada; 22 Faculty of Health, National Centre for Naturopathic Medicine, Southern Cross University, Lismore, NSW Australia

**Keywords:** Leadership program, Mentoring, Research capacity, Chiropractic, Early career researcher

## Abstract

The Chiropractic Academy for Research Leadership (*CARL*) was formed in 2016 as a global mentorship program for early career researchers in chiropractic. *CARL* is a three-year program anchored around three week-long residentials, alternating between Denmark, Australia, and Canada, with ongoing online activities and project work between residentials. This paper reports on activities and outputs of the second cohort of *CARL* Fellows, *CARL II*, that ran from 2020 to 2024. Despite disruptions from the global COVID pandemic, the *CARL II* cohort was successful in forming a tightly knit group of fourteen early career researchers from seven countries in Australia, Europe, and North America. *CARL* Fellows co-produced a range of research and leadership outputs, including 70 peer-reviewed manuscripts, and hosted two online conferences (*CARLoquium)* to support global research engagement during the pandemic. With the completion of the second cohort and recruitment of the third cohort completed, all underwritten by global financial support from chiropractic organizations, *CARL* is established as a global program building research capacity and academic critical mass in chiropractic.

**Supplementary Information:**

The online version contains supplementary material available at 10.1186/s12998-026-00641-3.

## Background

The chiropractic profession has faced significant challenges in establishing a robust research culture, essential for advancing evidence-based practice and improving patient care^1–3^. The lack of a mature research environment within chiropractic institutions is largely attributed to deficiencies in research capacity and leadership, exacerbated by the limited presence of chiropractic programs in major universities^4^. This challenge necessitates a concerted effort to build leadership and research capacity globally within the field, which is why we established the Chiropractic Academy for Research Leadership (*CARL*)—a global strategic initiative aimed at fostering research leadership and enhancing the evidence base for chiropractic practice^2^.

*CARL* is a competitive-entry program that aims to develop a critical mass of successful early career researchers through international networking and collaborations, all under the mentorship of senior academics. It is a three-year program anchored by yearly week-long residentials, self-directed online activities, and on-going project work. The residentials aim to provide dedicated and protected time from the day-to-day pressure of work environments, thus allowing for reflection and deeper discussions on advancing chiropractic and musculoskeletal research, academic leadership, personal and professional growth. The senior academic mentors in *CARL* also provide mentorship support and advice regarding individual research programs, strategic development, networking, and career pathways^2,5^.

The first *CARL* cohort consisted of 13 Fellows from seven countries and ran from 2017 to 2020. The visible output of *CARL I* was 38 scientific papers in 20 different journals, 81 conference presentations, professional advancement, and awards of many competitive grants. In these early years the *CARL* program soon became identified as a catalyst for career direction and growth, while fostering development of an effective and significant network of international collaborators in chiropractic^5^. The more invisible output was the personal value for the Fellows in terms of collegiality, friendship, and personal growth. As such, the *CARL* program demonstrated relevance and productivity that directly addressed both the challenge of fostering a mature international chiropractic research culture and infrastructure as well as the need for personal network and mentorship for early career researchers. Consequently, the three mentors in *CARL* decided to continue the program by recruiting a second cohort of Fellows, *CARL II*. This paper reports on the recruitment of the *CARL II* cohort, describes the selection of Fellows, the course and content of the program, summarizes the scientific and leadership activities and outputs, and finally reports on reflections from a workshop that aimed to capture the more intangible value of the program.

## Main text

In August 2019, the three mentors issued a call of interest for early career researchers, i.e., PhD students or those up to 3 years post-PhD completion, interested in joining the *CARL* program. Forty-one candidates from eight countries applied, of which 28 were selected for online interviews with the mentors. Following interviews, 14 Fellows from seven countries (Australia 4, Canada 3, Denmark 2, USA 2, Norway 1, Switzerland 1, UK 1) were selected and included in the *CARL II* cohort. The *CARL II* cohort officially joined the program in March 2020 and completed the program in August 2024 with the planned three-year program extended by two years due to COVID.

### Structure and activities in CARL II

#### COVID and CARLoquium

**Fig. 1:**
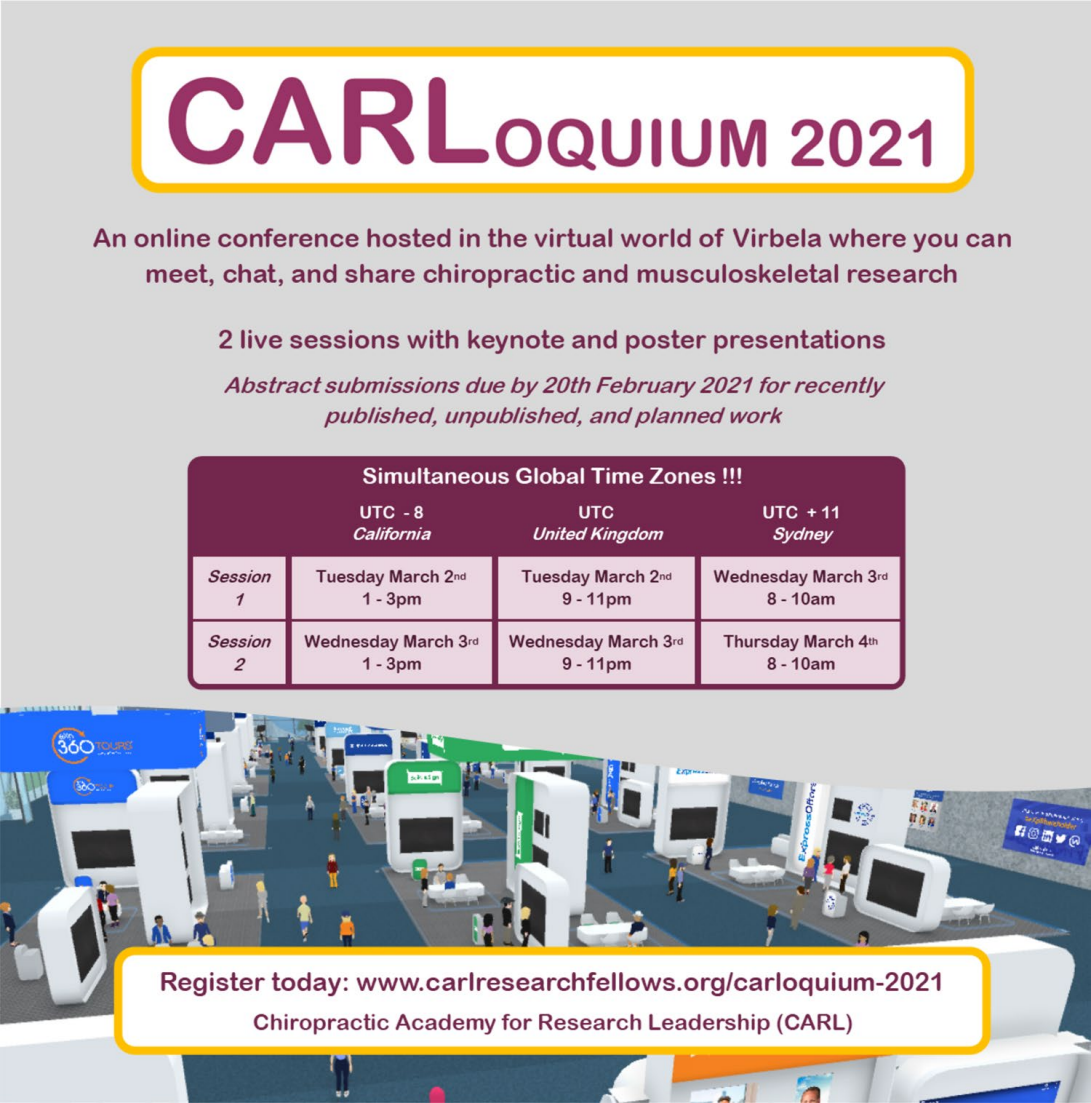
Advertising for the 2021 *CARLoquium*

The first residential for the *CARL II* cohort was scheduled for May 2020 in Sydney, Australia. However, global COVID-19 travel restrictions began in March 2020, forcing the program to shift to online meetings for interaction and project planning. During these early meetings, the Fellows proposed hosting an online international conference using a virtual platform that enables participants to create avatars and navigate a virtual conference center complete with auditoriums, poster halls, and meeting spaces. The resulting event, *CARLoquium*, was announced on social media, with a participation fee of 35 USD to cover software costs and prize funds (Fig. 1). Due to the pandemic restrictions continuing for nearly two years, two *CARLoquium* conferences were eventually conducted: the first in March 2021 and the second in March 2022. Each conference was a live two-day online event featuring keynote lectures and poster sessions, moderated and organized by *CARL II* Fellows. The 2021 conference accepted 108 abstract submissions, and the 2022 conference accepted 77 abstract submissions. The success of *CARLoquium* directly informed the platform and format of the *17th International Forum for Back and Neck Pain Research in Primary Care*, which was also held online in 2021 due to restrictions in international travel.

#### Residentials

Three week-long residentials were held for *CARL II*: Odense, Denmark in August 2022, Sydney, Australia in October 2023, and Edmonton, Canada in August 2024. All Fellows participated in all residentials except one who missed one residential. Residentials included guest speakers, workshops, worktime, and daily physical activity. Guest speakers represented successful early career and senior academics from different fields who presented and discussed diverse topics with the fellows such as career planning and trajectories, work-life balance in academia, equality in research, new strategies in academic publishing, research about management and leadership, and how to navigate social media and the press. A much valued and recurrent session throughout the residentials was “*Problem Solved”*. Here Fellows presented a professional or personal challenge within the ‘safe space’ of the *CARL* Fellowship, with the Fellow and wider group then exploring and providing reflections. This format helps provide fresh, useful perspectives and an opportunity to ‘share’ issues that may not normally be aired in a local work environment. The residentials also facilitated substantial periods for Fellows to undertake group work to develop and advance project design, data analyses, and manuscript writing. Finally, one daily session was collective physical activity that included walks, yoga, football (soccer), and zumba. To further encourage collaboration and discussion, regional *CARL* Senior Fellows, i.e., Fellows from previous *CARL* cohorts local to the region where the residential was held, were invited to join one day of the residential.

### Outputs

#### Scientific

*CARL II* was highly productive. The Fellows engaged in a wide range of scientific projects together, mainly based on existing data but also with regards to newly collected data and reviews of scientific literature. A *CARL II* publication was defined as a paper published in a peer-reviewed journal that included at least two Fellows with or without a mentor or a paper with at least one Fellow and at least one mentor. From 2020–2024, there were 70 peer-reviewed *CARL II* publications, and the Fellows presented 50 poster or podium presentations at national or international conferences. Outside of *CARL*, the Fellows collectively published a total of 171 peer-reviewed papers during the period of the program (Table 1).

**Table 1:** Scientific and leadership output, awards, degrees and promotions, and research grants for the *CARL II* cohort 2020 – 2024

*Scientific output*	
CARL papers*	70
Conference presentations	50
Non-CARL papers**	171
*Leadership output*	
Keynote talks, workshops, podcasts, committees, editorial	130
*Awards*	
Honorary or scientific	23
*Degrees and promotions*	
PhD degrees	9
Promotions	22
*Research grants*	
Research grants	63
Total amount €	21,226,510

*A CARL paper has two CARL II Fellows or one Fellow and one mentor as authors

**A non-CARL paper has only one CARL II Fellow as author

#### Leadership activities and awards

The *CARL II* Fellows undertook numerous new leadership activities while supported by the *CARL* program including presenting national and international conference keynote talks, leading workshops or moderating sessions at conferences and meetings, participating in academic and research podcasts, accepting invitations to become members of scientific and professional committees and boards, directing courses, and undertaking editorial work for scientific journals. Collectively, *CARL II* Fellows engaged in 130 leadership activities during their time on the program. *CARL II* Fellows received a total of 23 awards including honorary awards and awards for scientific presentations (Table 1).

#### Degrees, promotions and grants

Nine fellows were awarded their PhD degree during the *CARL II* period. In addition, 22 promotions or appointments to new academic positions were achieved across the *CARL II* cohort during the program. Collectively, *CARL II* Fellows were awarded 63 competitive research grants from a range of funding bodies as primary- or co-investigators for a total value of € 21,226,510 during their time on the program (Table 1).

#### Workshop about importance of CARL

During the Edmonton residential (2024), a half-day workshop about the deeper meaning of *CARL*, what *CARL* has meant for the Fellows personally, and the perceived impact on the chiropractic profession was held. Four North American Senior Fellows (Funabashi, Pohlman, Pagé, De Carvalho) joined the *CARL II* Fellows for this workshop, led by a Canadian expert in learning program design, development, and delivery (https://www.flip-learning.ca/). Mentors and Fellows were divided into three groups with each group tasked with producing an image and a statement that communicated the output from their discussions (Table 2).

**Table 2:** Statements recorded by the three groups of *CARL II* and North American senior *CARL* Fellows during a workshop at the edmonton residential in 2024

Group 1 statement	Group 2 statement	Group 3 statement
The *CARL* program gave me a unique opportunity and space to grow professionally, collaborate internationally, and become a research leader in chiropractic The deep friendships gained through *CARL* have been life changing	*CARL* transformed me as a researcher and as a person It made me learn and think about things I didn’t necessarily think were possible Through the experience of *CARL*, I have grown and sustained a network of collaborators I really enjoy working with *CARL* demonstrated the importance of, and skills to cultivate, a thriving and joyful work-culture I attribute my tenured position to the skills and connections I built as a *CARL* Fellow I feel more confident to tackle complex research problems because of my own development and the backup in the group	*CARL* provided the community that drove my career forward *CARL* provided the community that continues to drive and fuel my career and personal growth The chiropractic profession is in a better place because of *CARL* Leave the campground in a better shape than when you found it

Detailed list of outputs from the *CARL II* cohort can be found in Online Appendix 1.

## Discussion

Despite initial challenges during the global COVID lockdown, *CARL II* was a highly successful and productive cohort. Research and leadership outputs combined with academic professional advancement, scientific and professional awards, and award of substantial grant funding establish the *CARL II* Fellows as part of the future global scientific leadership in chiropractic. Importantly, with completion of the second cohort, the *CARL* mentorship program has become established as a key piece of infrastructure in building global chiropractic academic critical mass for the future.

Mentorship programs can significantly enhance the career development of healthcare researchers by offering a collaborative and supportive network, tailored guidance, and support. Frei et al. reviewed 14 studies dealing with academic mentorship programs in healthcare and concluded that they were beneficial particularly for those inclined towards research careers, thereby emphasizing mentorship's role in guiding strategic career choices^7^. Mentorship programs can foster the development of essential professional behaviors and skills vital for a successful career in healthcare research through improvements in collaboration and interprofessional teamwork, which are core elements in *CARL*^8^. Importantly, mentorship programs not only enhance individual competencies but also serve to bolster institutional and profession-related research outputs by higher retention rates and higher rates of research productivity among people who have been mentorship program participants^9,10^. This underscores the potential of mentorship programs such as *CARL* to not only enhance individual researcher capabilities but also to foster a collaborative research culture within an institution and a profession.

A regular theme of discussion among *CARL* II Fellows and the mentors was *diversity, equity, and inclusion* in research and the chiropractic profession. Discussions about gender issues were particularly prominent. Therefore, *CARL* is proud that 46% of *CARL* I Fellows and 50% of *CARL* II Fellows are women. Most keynote speakers at both *CARLoquium* conferences were women, and in 2022, poster presenters were 47% women (gender data not collected in 2021). The *CARLoquium* conference data outperforms the data on gender diversity at other chiropractic research conferences between 2010 and 2019, highlighted in a 2023 *CARL* II publication^6^.

Mentors for the first two cohorts were male-only, however, for *CARL* III a senior female chiropractic academic (Diana De Carvalho) has been included as a mentor.

The chiropractic profession still faces significant challenges in cultivating a robust research culture, necessary for the progression of evidence-based practice and the integration of chiropractic care within broader healthcare systems. One of the most prominent challenges stems from the differing views as to the importance of research and evidence within the chiropractic profession, which impedes unified efforts towards advancing research initiatives and even creates a hesitance within some groups in chiropractic to adopt evidence-based standards of care^11,12^. Exacerbating these challenges, current chiropractic research environments are hampered by limited resources and an absence of structured mentorship programs, resulting in a cycle where inadequate research activity fails to stimulate growth within the chiropractic research community^4^. *CARL* addresses these challenges directly, and the broad financial support for *CARL* from chiropractic organizations globally reveals a desire by chiropractic political leaders to improve the academic critical mass within chiropractic. This alone illustrates the need for and viability of *CARL*, evidence of which are the secured resources globally to support a *CARL III* cohort, consisting of 16 new fellows selected through a competitive process. Of note, one of the CARL Mentors (Jon Adams) is founder and mentor in similar programs for other health professions fx the SOLAR program in osteopathy that follows a similar framework^13^. Future developments in structure and funding models will likely be inspired between these networks.

A few months after completion of the last residential for the *CARL II* cohort and shortly after recruitment of *CARL III*, one of the founders and mentors, Professor Greg Kawchuk, became critically ill and passed away. Professor Kawchuk’s contribution to *CARL* as a mentor and friend for *CARL I* and *CARL II* Fellows as well as a very visible advocate for academic development in chiropractic and evidence-based patient care cannot be overestimated. He is dearly missed. Professor Diana De Carvalho, herself a *CARL I* Fellow, has been selected as a new mentor in *CARL*.

## Conclusions

The Chiropractic Academy for Research Leadership, *CARL*, has established itself as a vehicle for building global academic capacity in the chiropractic profession. The second cohort of *CARL* Fellows, *CARL II*, was highly productive in terms of scientific and leadership outputs and many Fellows completed degrees and achieved academic promotions. In addition, the personal value of *CARL* for individual careers were highlighted by the Fellows. Funding for *CARL III* has been secured, and the new cohort of Fellows have been recruited.

## Supplementary Information

Additional file1 (XLSX 69 kb)

## Data Availability

Not applicable.

## Abbreviations

CARL: Chiropractic Academy for Research Leadership
COVID: Coronavirus disease
